# Pre-harvest mango yield prediction using artificial neural networks based on leaf nutrient variability

**DOI:** 10.7717/peerj.20013

**Published:** 2025-09-16

**Authors:** Abdullah Alebidi, Khalid F. Almutairi, Rashid S. Al-Obeed, Essa Makhasha, Abdulwahed M. Aboukarima, Mahmoud Abdel-Sattar

**Affiliations:** 1 Department of Plant Production, College of Food and Agriculture Sciences, King Saud University, Riyadh, Saudi Arabia; 2 Department of Agricultural Engineering, College of Food and Agriculture Sciences, King Saud University, Riyadh, Saudi Arabia

**Keywords:** Chlorophyll, Carbohydrate, C/N ratio, Nutrient, Prediction, Productivity, Sustainable

## Abstract

Accurate pre-harvest mango yield prediction provides valuable insights for improving productivity, reducing food waste, enhancing food security, and supporting the farmer livelihoods. In this respect, nine mango orchards, which had different agricultural practices, were selected to attain the essential data to integrate yield and leaf nutrient variability using an artificial neural network model. In the mango leaves, noticeable variations were detected in concentrations of nitrogen (N), phosphorus (P), potassium (K), magnesium (Mg), calcium (Ca), chlorophyll *a* (Chl *a*), chlorophyll *b* (Chl *b*), and total carbohydrates (Carbs) fraction. The yield variation between seasons is high; the ON season gave a high yield, and the OFF season gave a low yield. The results revealed that at the pre-harvesting time, the relationship between the carbohydrate: nitrogen (C/N) ratio and the yield against the growing season had a reverse trend. The artificial neural network (ANN) mango yield model was created using eight inputs representative of the nutrient status of leaves. The ANN model achieved an accurate match in predicting mango yield from investigated parameters, with an R^2^ value of 0.975 using a testing dataset, and the mean absolute percentage error (MAPE) was 3.02%. The concentration of Chl *a*, Chl *b*, and the Carbs fraction had the greatest contribution in predicting mango productivity. It was concluded that the ANN model performed adequately and captured the non-linear effects of the interaction between the nutrition status of the mango leaves and mango productivity.

## Introduction

Mango (*Mangifera indica* L.) is a vital commercial fruit crop full-grown in tropical and sub-tropical portions of the world (Bompard, 2009; Nafees et al., 2010). It is sometimes referred to as the “king of fruits”. The crop offers employment to several societies, from local growers to laborers within the manufacturing and supply chain (Arcila-Diaz, Mejia-Cabrera & Arcila-Diaz, 2024). Mangoes are currently grown in over 100 nations worldwide (Mitra, 2016), which are located between 30° north and 30° south latitude (Liang et al., 2024). However, mango cultivation has significantly promoted growers’ income in its production regions (Liang et al., 2024). By way of 40 metric tons of mangoes are produced worldwide, and they are an important part of many people’s lives because they are a rich source of nutrients and a source of income for millions of people (Mitra, 2016). Additionally, the nutritional value and variety of phytochemicals that the mango contains make it one of the most significant fruits in the world (Yahia et al., 2023). Moreover, it is widely consumed in fresh and processed forms and appreciated for its high nutritional value and health benefits, such as carbohydrates, lipids, protein, fatty acids, organic acids, vitamins, macroelements, microelements, and volatile constituents (Jahurul et al., 2015; Abdel-Sattar et al., 2024). Furthermore, mangoes are a good basis of ascorbic acid, phenolic compounds, carotenoids, and other dietary antioxidants “(bioactive compounds)”(Lebaka et al., 2021). This tropical fruit isn’t best valued for its specific taste and dietary advantages; however, it additionally performs an essential function in producing earnings and offering financial balance in diverse areas across the world. Mango manufacturing is an agricultural hobby that represents a large supply of earnings for farmers (Arcila-Diaz, Mejia-Cabrera & Arcila-Diaz, 2024). The optimum advance temperature for mango is 24–27 ^∘^C, and it is not accepting to cold, supporting damage below 0 ^∘^C (Normand, Lauri & Legave, 2015).

Saudi Arabia has an area of 2,150,000 km^2^ in the Arabian Peninsula. More than 35 million people live there. The country is distinguished by its arid climate and a really dry climate (Morgounov et al., 2022). The weather in the Kingdom of Saudi Arabia fluctuates from region to region due to the different topography. Due to the harsh climate, agriculture in particular, crop cultivation, is only possible in limited areas (Elzaki, Elrasheed & Elmulthum, 2022). The focus of agricultural production in Saudi Arabia is primarily on self-sufficiency. However, as part of its Vision 2030, the Kingdom of Saudi Arabia is focusing significant attention on the agriculture sector to ensure food supply for its growing population and to export any surplus (Elzaki, Elrasheed & Elmulthum, 2022; Rahman et al., 2022). Furthermore, to address climate change, population expansion, and sustainable development goals, the agriculture industry must be sustainable and efficient (Martos et al., 2021). Accordingly, many agricultural programs originated at diverse times to confirm food security and rural development (Bailey & Willoughby, 2013). However, horticulture—which deals with the cultivation and enhancement of crops—is an essential branch of agriculture (Parmar et al., 2023). As said by the Ministry of Environment, Water and Agriculture: Riyadh, Saudi Arabia (2020), most of the arable land is irrigated. The date palms are grown on around 152,704 ha; olives on 30,960 ha; and fruit trees on 24,705 ha. These data indicate that horticulture crops are important for the national economy of Saudi Arabia (Alshammari et al., 2022). Furthermore, in accordance with the aims of Vision 2030, Saudi Arabia’s yearly mango output rose to 88,600 tons, representing 60% self-sufficiency, despite mangos being a substantial fruit crop extensively grown throughout the Kingdom of Saudi Arabia (Achard, 2023). Additionally, mango is one of the economically feasible tropical crops in Saudi Arabia, with the production period from April to August. According to Abdelbaki & Alzahrani (2024), the cropping shape in Saudi Arabia included a varied range of 39 crops. These crops together occupy a land area of 744,536 ha and yield a total production of 10,548,311 tons. The cultivated area of mango was 6966 ha, with a productivity of 89.5 × 10^3^ tons and a rate of 12.8 tons/ha (Abdelbaki & Alzahrani, 2024).

The production efficiency of mango orchards varies with climatic conditions such as temperature, humidity, solar radiation, rainfall, and cultural management (Dhonju et al., 2024). Additionally, it fluctuates according to plant nutrition, number, type of additional foliar applications and method, amount of irrigation, quantity, disease, and pest management (Dhonju et al., 2024). Moreover, it differs according to plant dynamics such as age, crop load, and vigor, stress between seasons, and between orchards (Dhonju et al., 2024). These differences in production efficiency are reflected in the nutritional status of mango trees, but the main causes of low mango productivity are often related to nutrition (Alebidi et al., 2023). Plant leaves are the primary site of metabolism, producing photosynthates (Barker & Pilbeam, 2007; Lahiji et al., 2018). Other parts of the plant receive the photosynthates. Accordingly, various qualitative characteristics and fruit crop output are linked to nutrient concentrations in the leaf (Torkashvand, Ahmadipour & Mousavi Khaneghah, 2020). Nutrition is the most important among the many problems affecting growth and fruit yield (Ferguson et al., 2003; Gee et al., 2018). Accordingly, variations in nutrient availability are reflected in the mineral structure of the leaves (Torkashvand, Ahmadipour & Mousavi Khaneghah, 2020). The stability and availability of nutrients in plant leaves significantly impact the quantity and quality of fruit produced (Huang & Ferguson, 2003; Lahiji et al., 2018). However, Awasthi et al. (1988) stated a direct correlation between leaf nutrients and the yield and quality of apples, while Lahiji et al. (2018) informed an important correlation between leaf nutrient concentrations and olive yield, as well as Torkashvand, Ahmadipour & Mousavi Khaneghah (2020) used the artificial neural network (ANN) model to predict kiwifruit yield based on the leaf nutrient concentration.

Yield prediction methods can be classified as either direct (counting fruits on trees) or indirect (application of mathematical models using relevant features) based on the input features (*e.g.*, weather, orchard management, vegetation indices, *etc*.), estimation platform (vehicle mounted, unmanned aerial vehicle platforms, and satellite, ground), and other factors (He et al., 2022). Timely and precise pre-harvest crop yield estimates are necessary to ensure harvesting, storage, transportation logistics, and effective marketing (Anderson et al., 2021). However, statistical forecasting aids in decision-making and planning the future more efficiently and professionally (Rathod & Mishra, 2018). Forecasting is a main piece of the emerging economy, so that appropriate arrangements can be made for the sustainable development of the country (Rathod & Mishra, 2018). Today, modern prediction methods are increasingly used (Moriarty, 2023). However, crop yield prediction is not a trivial task but comprises several complicated stages. Thus, recently, the application of computational intelligence and machine learning methods has been increasingly recognized in crop yield estimation. However, few studies have applied such computational methods to estimate fruit yield. Thus, fruit yield prediction models are developed for mango crops (Payne, Walsh & Subedi, 2016; Kumari et al., 2022). However, the use of new technology in agriculture has grown imperative (Neethi, Kiran & Tiwari, 2023), and it has different topics in mango yield predictions, such as using image analysis and machine learning algorithms for the estimation of mango fruit production (Arcila-Diaz, Mejia-Cabrera & Arcila-Diaz, 2024), intelligent mango canopy yield estimation (Neethi, Kiran & Tiwari, 2023), mass estimation of mango fruits (cv. Nam Dokmai) by linking image processing and artificial neural networks (ANN) (Utai et al., 2019), and using random forest modeling for the estimation of mango (cv. Chok Anan) fruit yields (Fukuda et al., 2013). However, because of their high predictive capability, ANNs have a high potential for modeling nonlinear systems in yield prediction (Kumari et al., 2022; Bharti Das et al., 2023). ANN is a nonlinear data-driven method that tracks a self-adaptive learning style (Das, 2019). ANN discovers relationships by examining many input and output cases, creating equations that can be used for prediction. Developing models using ANN does not require prior familiarity with the inputs and outputs. ANNs are superior to other linear models because they can better determine the optimal patterns of variables and have fewer inaccuracies (Abdipour et al., 2019). These advantages make ANNs popular in various fields, particularly crop yield prediction (Al-Adhaileh & Aldhyani, 2022; Sajindra et al., 2023).

With these advantages of ANN models, this research aims to contribute to the development of sustainable mango production systems by assessing the impact of variation in the leaf nutritional status of mango trees on pre-harvest mango yield. This research also, aims to predict fruit yield in mangoes *via* a new ANN modeling approach, using explores orchards-to-orchards variability in leaf nutritional status for ‘Timor’ mango trees sampled under different culture practices of individual trees across orchard blocks. The novelty of this study is the investigation of the estimation of mango yield towards a more sustainable supply of fruit yield. This work also determines the contribution of affecting input variables on mango yield to manage their effects for improving production and efficient use of farm inputs and resources.

## Materials & Methods

### Experimental location and mango trees description

Data was sourced from nine (*Mangifera indica* L. cv. Timor) orchards in the season of 2023 (ON season) and the season of 2024 (OFF season). Orchards were located throughout the significant mango-producing areas in the Jazan region of southwest Saudi Arabia. Situated in the southwest of Saudi Arabia, the 13,457 km^2^ Jazan region is distinguished by its unique physical, environmental, and cultural variety (Islam et al., 2025). Jazan region is located in the southwest corner of the Kingdom of Saudi Arabia between longitude 42.7076°E and latitude 17.4751°N (Al-Mekhlafi et al., 2021). The climate of Jazan is characterized by arid and dry conditions typical of a desert region (Hasanean & Almazroui, 2015). Temperatures in Jazan remain consistently high throughout the year, accompanied by moderate humidity levels (Aldhafiri, Ali & Labban, 2025). The temperatures range from 31 °C in January to 40 °C in July. Despite this, Jazan has a low annual rainfall, averaging about 162 mm (Islam et al., 2025).The tree in the experimental fields was eight years old, budded on Kutchineer seedling rootstocks set apart 8 × 8 m in sandy soil and grown under different cultural practices. However, the selected trees for gathering data were healthy, uniform, and defect-free. The drip irrigation was applied using two laterals per row; one 0.5 m from each side was used for irrigation purposes the row, and drippers (4 L/h) were located one meter apart along the laterals. The orchards selected for data collection were consistent in planting date and cultivars but differed from one orchard to another in the applied agricultural practices, such as the amount and frequency of irrigation, the amount and type of nutrition and fertilization dates, the time and amount of pruning, and how pests and diseases were managed.

In this study, the leaves and fruits were taken from nine mango orchards with nine replicates per orchard and one tree per replicate (*i.e.,* nine orchards × nine replicates × one tree per replicate = 81 samples). When the fruits ripened, they were harvested from each replicate at the start of the last week of April in both seasons. All the fruit samples from each tree were picked, and the total yield of the trees was calculated by multiplying the weight of fruits per tree (kg) and then converted to tons/ha. The same trees were monitored in both seasons.

### Leaf nutritional status measurements

To ascertain the nutritional status of the trees, twenty mature leaves from the pre-labeled shoots, including the blade and petiole, from the terminal portion of the non-fruiting branch of the 6–7-month-old branch (the fourth and fifth newly mature leaves) were collected according to the method described by Chadha, Samra & Thakur (1980) and Makhasha, Al-Obeed & Abdel-Sattar (2024) four weeks before harvest. The Li (2006) method was used to analyze the chlorophyll content (Chl *a* and Chl *b*). A subsample of fresh leaves was promptly cleaned with water, and 1 g of them was powdered in liquid nitrogen in a mortar that had been chilled. They were then homogenized for 30 s in 5 ml of 80% acetone, and the extract was filtered. Using a spectrophotometer, the optical density of a given filtrate volume was determined at wavelengths of 645 nm for Chl *b*,and 663 nm for Chl *a*. Sumanta et al. (2014) provided equations for calculating concentrations of Chl *a* and Chl *b* using a spectrophotometer (µg/ml), which were subsequently translated to (µg/g fresh mass) (µg/g FM).

A spectrophotometer was used to measure the leaves’ total carbohydrate content using the Somogyi (1952) technique at a wavelength of 490 nm. The mean values were then reported as a percentage. The leaf samples were cleaned with tap water, rinsed twice with distilled water, oven-dried at 70 °C until their weight remained constant, and then finely crushed in accordance with Makhasha, Al-Obeed & Abdel-Sattar (2024) in order to ascertain the leaf mineral contents of the samples. A 250 mL digestion flask that had been cleaned with acid and distilled water was filled with 0.2 g of pulverized plant material. Six milliliters were mixed with five milliliters of concentrated sulfuric acid and one milliliter of 70% perchloric acid in a 5:1 (v/v) ratio. The samples were digested over an electric heater until dense white fumes developed and the solution turned clear, yielding a volume of around 2.5 mL. The samples were quantitatively transferred into a 50 mL volumetric flask after cooling, and they were then diluted with distilled water. The distilled water was used to get the required volume. Using the micro-Kjeldahl method as outlined by Chapman & Pratt (1978), the digested solution’s nitrogen (N) and phosphorus (P) contents (%) were ascertained as described by Evenhuis & De Waard (1980). The methods of Evenhuis, (1976) and Murphy & Riley (1962) were used to determine the total N and P calorimetrically using a spectrophotometer (9100UV–VIS, Manufacturer: PerkinElmer, Woodbridge, ON, Canada). As Jackson (1967) explains, flame photometry (A & E-FP8501, A& E Lab (UK) Co., Ltd., London, UK) was used to measure the potassium (K) concentration. Using ionic chromatography plasma spectroscopy (Optical Emission Spectrometer; Perkin Elmer, Woodbridge, ON, Canada), the calcium (Ca) and magnesium (Mg) concentrations were ascertained in accordance with Donohue & Aho (1992) approach. By dividing the leaf’s total carbohydrate content by its total nitrogen content, the carbohydrate: nitrogen ratio (C/N) was calculated.

### ANN model for mango yield prediction

Two more steps should be taken into consideration after data collection and before ANN training: pre-processing the data and dividing the input data into sub-set groups. The ANN model can be used more effectively if the pre-processing operation is performed on input and output data. The software Qnet v2000 for Windows, established by Vesta Services Company, was directed to achieve normalization between 0.15 and 0.85. The normalization preprocessing steps are involved in previous studies; however, the equation for normalization input and output data between 0.15 and 0.85 is described by Naji Al-Dosary et al. (2023). The software returned the data to the original form when the predictions were finished. To test its judgments, Qnet v2000 was also directed to choose a reasonable number of points at random. However, Qnet v2000 was preferred to run the simulation due to it is a graphical user interface simulation software and relies on the famous typical backpropagation learning algorithm. Furthermore, the single-layer feedforward neural network has been documented for its effectiveness as they are one of the most commonly used fast-learning algorithms (Marzouq et al., 2019). Furthermore, feedforward neural network models are easy to use and can signify quantifiable functions with high accuracy, particularly regarding weather forecasting, so they are being researched (Marzouq et al., 2019; Bounoua & Mechaqrane, 2021). Furthermore, it has been confirmed that a single-layer feedforward neural network can solve various regression problems (Bounoua & Mechaqrane, 2023). In this study, a three-layer neural network architecture was formed using viable software called Qnet v2000 for Windows, established by Vesta Services Company.

A typical backpropagation learning algorithm was employed to forward the ANN structure, and mango yield was predicted through supervised training. Usually, this kind of neural network comprises numerous layers of connected neurons. One or more hidden layers, referred to as the network’s first and last layers, may exist between the input and output layers. The process for creating and evaluating a neural network model with Qnet v2000 can be found in Al-Sager et al. (2024). Back-propagation training involves multiplying the input data by the weight, adding and accumulating the bias, and then entering the result—the nerve’s input—into the transfer function. After that, the output neuron entered the output layer after being computed using transfer functions. This layer goes through the same process: the linear transfer function’s output is compared to the predicted value, and the error value is determined. In order to achieve the ideal number of hidden neurons through trial and error, the backpropagation algorithm corrects the weights and bias values if the error value exceeds the specified value. This procedure is continued until the error value falls below the specified value.

To avoid the algorithm’s hidden bias for higher values in the dataset, normalization was carried out across the input and output of the dataset (Qazi et al., 2015). Mango yield was the output of the ANN model, which included some variables as input variables, as confirmed in Table 1. These variables included leaf N, Mg, P, K, Ca, Chl *a*, Chl *b*, and Carbs fraction at harvesting time.

**Table 1:** Some of the gathered data for constructing an ANN model is recognized to predict the mango yield.

**Independent variables (inputs)**								**Dependent variable (output), yield**
**N**	**P**	**Mg**	**K**	**Ca**	**Chlorophyll content a**	**Chlorophyll content b**	**Total carbohydrates fraction**	
**(%)**	**(%)**	**(%)**	**(%)**	**(%)**	**(mg/g FM)**	**(mg/g FM)**	**(%)**	**(ton/ha)**
1.97	0.34	0.98	2.71	0.78	1.92	1.27	24.96	8.15
1.60	0.22	0.81	2.21	0.48	1.49	0.84	14.72	5.69
1.35	0.16	0.56	1.96	0.27	1.15	0.62	10.56	4.73
1.83	0.32	0.93	2.57	0.72	1.76	1.15	21.44	7.40
1.77	0.30	0.90	2.43	0.64	1.67	1.03	18.88	6.67
1.38	0.14	0.57	2.01	0.27	1.25	0.69	7.68	8.93
1.63	0.22	0.85	2.27	0.55	1.44	1.00	14.08	10.67

**Figure 1:**
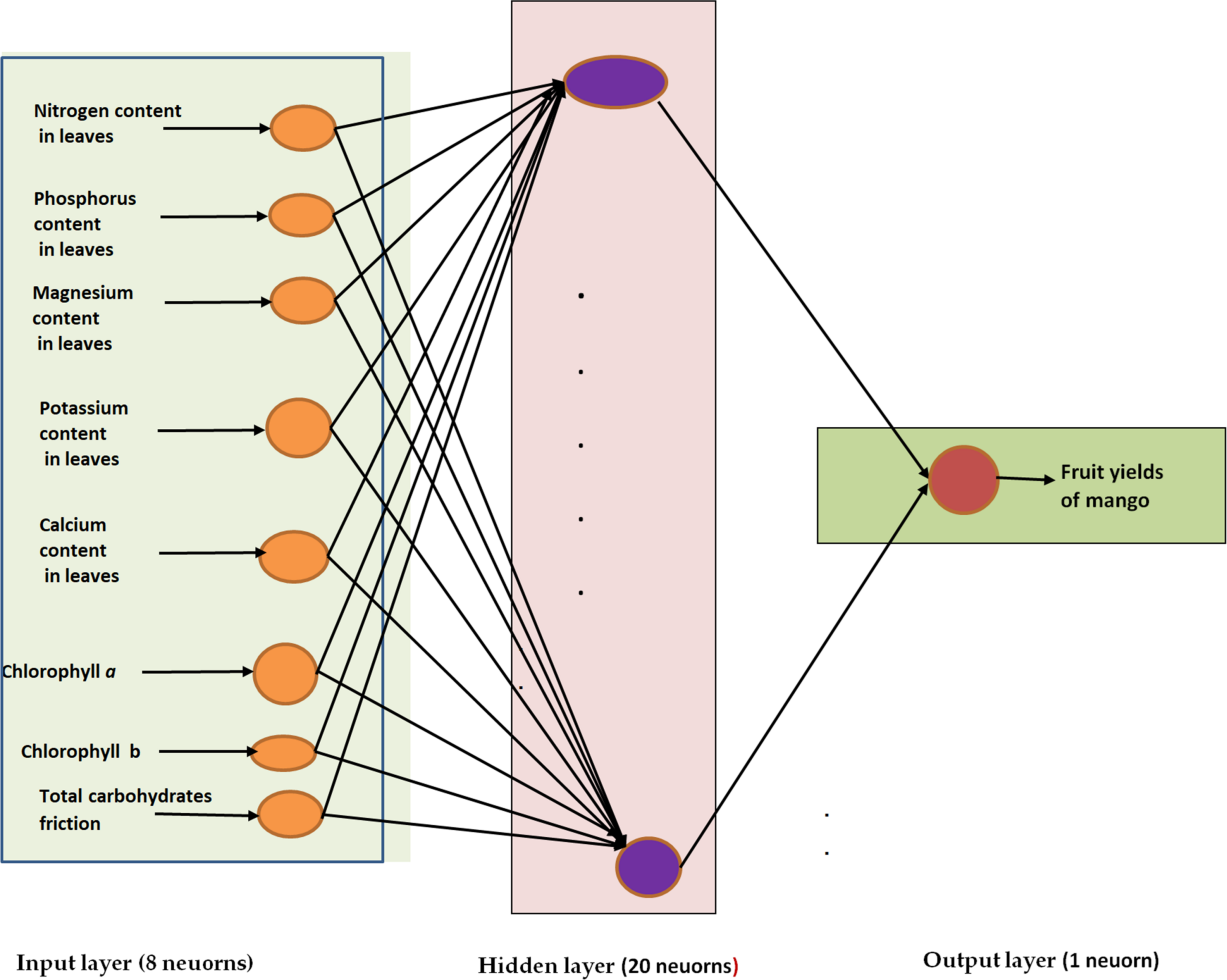
The optimal ANN prediction model structure (8-20-1) for mango yield.

There were two datasets used: 130 samples for training and 32 samples for testing. The ANN model was constructed, weights and biases were established, and the model was optimized using the training dataset. According to Al-Sager et al. (2024), this study investigated several neural network topologies while accounting for various variables, including the kind of activation function, the number of hidden layers, and the number of neurons in each hidden layer. The optimal ANN prediction model structure, 8-20-1 (Fig. 1), with sigmoid activation function (0,1) (Balaji & Baskaran, 2013), was obtained through trial and error. In the work of Kassem et al. (2023), the appropriate number of hidden layers and their neurons were fixed through a process of trial and error. After 100,000 cycles, the training procedure was completed, resulting in a test error of 0.029685 and a training error of 0.0222782. The momentum coefficient was 0.8, and the learning rate was 0.026515.

The coefficient of determination or R-squared (R^2^), mean absolute percentage error (MAPE), mean absolute error (MAE), and root mean square error (RMSE) were hand-me-down to assess the ANN model:

(1) \begin{eqnarray*}MAPE=100\ast \frac{1}{Ntt} \ast \sum _{q=1}^{Ntt} \left\vert \frac{{P}_{q}-{\hat {P}}_{q}}{{P}_{q}} \right\vert \end{eqnarray*}

(2) \begin{eqnarray*}MAE= \frac{\sum _{q=1}^{Ntt} \left\vert {P}_{q}-{\hat {P}}_{q} \right\vert }{Ntt} \end{eqnarray*}

(3) \begin{eqnarray*}RMSE=\sqrt{ \frac{\sum _{q=1}^{Ntt}{ \left( {P}_{q}-{\hat {P}}_{q} \right) }^{2}}{Ntt} }\end{eqnarray*}

where ${\hat {P}}_{q}$ is the forecast value, P_q_ is the experimental value, and Ntt is the total number of data points in the test and training datasets. According to Qazi et al. (2015), a good forecast has been made if the MAPE value is between 10% and 20%.

### Relative importance of parameters related to ANN model for mango yield prediction

Sensitivity analysis determines which input factors have the most effect on the ANN model’s results (outputs) (Yousefi et al., 2021). Sensitivity analysis is the training of examination in which input parameters have the most important impact on the results (outputs) of the ANN model (Yousefi et al., 2021). The sensitivity analysis permits each neural network to display which input parameters are most vital and have the highest influence on the explained variables (Mrzygłód et al., 2020). However, the sensitivity analysis in this study evaluated the significance of several effective independent variables, including leaf N, P, Mg, K, Ca, Chl *a*, Chl *b*, and Carbsfraction at the harvesting stage, in the ANN model of predicting the mango yield. The Qnet v2000 method (Vesta Services Inc., 2000), which is presented by Al-Dosary, Alnajjar & Aboukarima (2023) was employed to determine sensitivity analysis by calculating contribution percentage to identify the most beneficial variable in the ANN model.

### Results and Discussion

#### Analyzing leaf nutritional status

To monitor and control the nutrient requirements of the plant, it is necessary to analyze its leaves. Because the presence of nutrients in the soil under the right circumstances does not always ensure that these elements are absorbed, the mineral analysis of the leaf helps determine whether there are disruptions in mango nutrition (Faria et al., 2016). Nutrient deficiencies and excesses can be determined by visual leaf symptoms (Khan & Ahmed, 2020). The status of nutrient indices is often indicated by leaves at different times for different minerals (Khan & Ahmed, 2020). In this study, the mature leaves were used to measure the nutritional status of the trees. Orchard-to-orchard variations were observed in leaf N, P, Mg, K, Ca, Chl *a*, Chl *b*, and Carbs fraction at the harvesting time. Table 2 shows the minimum, maximum, standard deviation, and average leaf nutrient status of the selected mango trees (cv. Timor) orchards (two seasons).

**Table 2:** Minimum, maximum, standard deviation, and average of Timor mango leaf nutrient status (two seasons).

	**Leaf nutrient status at pre-harvesting time**									**Yield**
**Statistical criteria**	**N**	**P**	**K**	**Ca**	**Mg**	**Chlorophyll a content**	**Chlorophyll b content**	**Total carbohydrates** **Fraction**	**C/N ratio**	
	**(%)**	**(%)**	**(%)**	**(%)**	**(%)**	**(** **mg/g FW)**	**(mg/g FW)**	**(%)**	**(-)**	**(tons/ha)**
	**ON season**									
Minimum	1.30	0.11	0.45	1.92	0.21	1.14	0.62	6.40	4.89	8.26
Maximum	1.85	0.33	0.96	2.57	0.75	1.65	1.23	20.48	11.13	12.02
Average	1.57	0.21	0.77	2.22	0.47	1.39	0.92	12.58	7.80	10.23
Standard deviation	±0.17	±0.07	±0.16	±0.20	±0.18	±0.16	±0.19	±4.57	±2.06	±1.16
	**OFF season**									
Minimum	1.32	0.15	0.55	1.94	0.26	1.13	0.61	9.92	7.35	4.66
Maximum	1.99	0.39	0.99	2.73	0.80	1.92	1.31	25.28	12.77	8.17
Average	1.68	0.26	0.82	2.33	0.56	1.56	0.96	17.31	10.13	6.37
Standard deviation	±0.21	±0.07	±0.13	±0.25	±0.17	±0.25	±0.23	±4.96	±1.69	±1.10

As seen in Table 2, there were variations in leaf nutrient status in two seasons. Faria et al. (2016) found similar variations for mango leaves (cv. Tommy Atkins) at the flowering and fruiting stages, as well as Musfiq-Us-Salehin et al. (2020) reported variations in the leaf nutritional status of mango fruit. These differences in leaf nutritional status may be attributed to growth stages and the production cycles independently. Furthermore, the differences in leaf nutritional status may be due to differences in soil nutrient availability, soil type, and growing conditions (Faria et al., 2016), as soil reaction settings, salinity, or antagonism between nutrient elements can cause unwanted fluctuations in nutrient absorption.

Nitrogen (N) is an important nutrient in mango, influencing both productivity and fruit quality (Bally & Still, 2013). Tree growth and yield were very poor when N was not applied and with very high N doses (Reddy et al., 2000). However, Silber et al. (2022) reported that N is a vital nutrient that influences numerous parameters of mango production, such as vegetative growth, alternate bearing, photosynthesis, quality of shoot bearing and panicles, embryo abortion, fruit color, and anthracnose disease.

**Figure 2:**
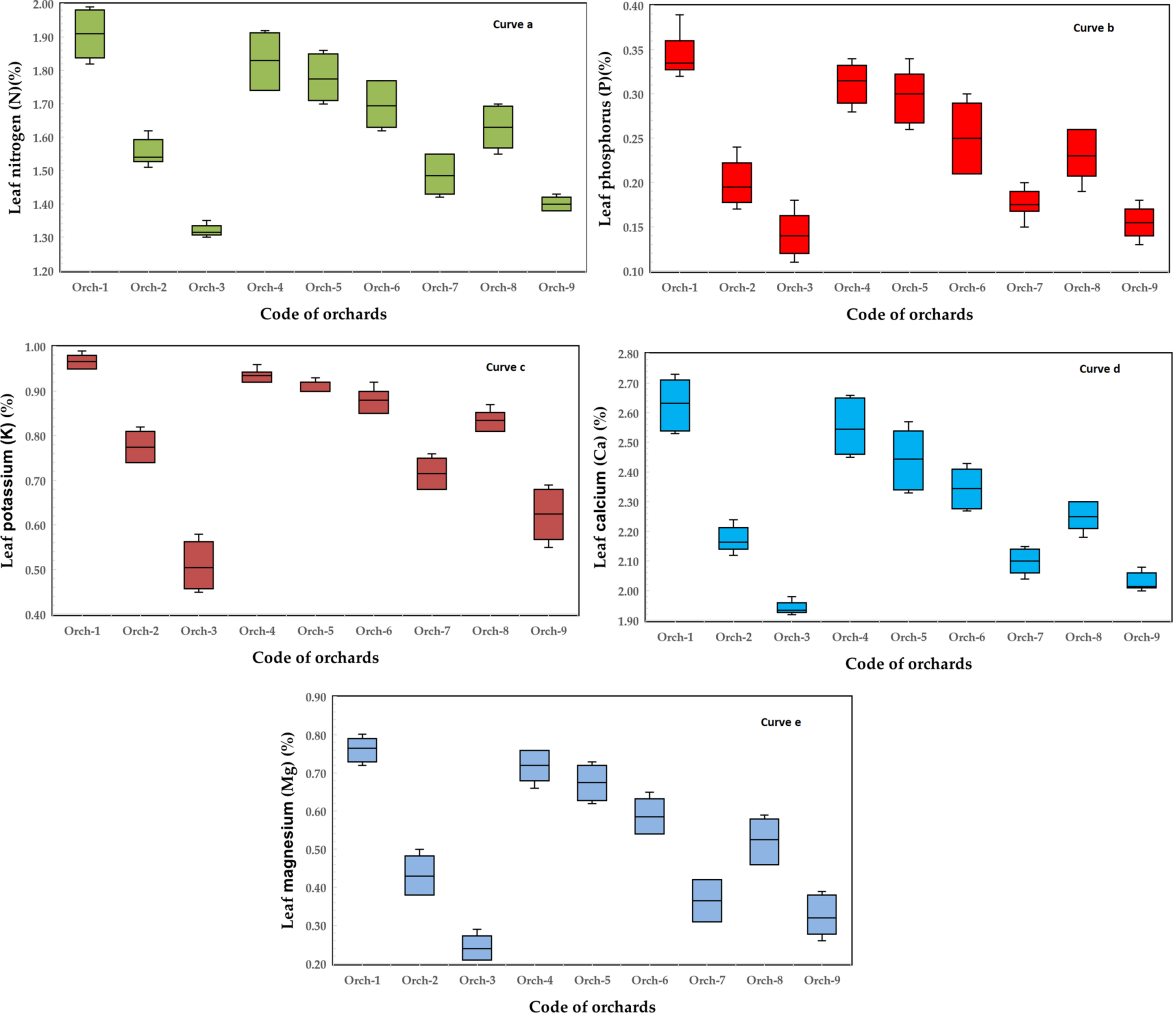
Variation of nitrogen, phosphorus, potassium, calcium, and magnesium in a leaf of the selected trees of mango (cv. Timor) orchards (two seasons) (data collected from nine orchards).

The results show that the leaf nitrogen range was 1.32–1.99% with an average of 1.68 ± 0.21% in the OFF season and 1.30–1.85% with an average of 1.57% ± 0.17% in the ON season (Table 2) and with the comparison with the results of Reddy et al. (2001), who presented mean leaf nutrients of nitrogen form different orchards irrespective of variety (1995–1997) of 1.66–2.02% with mean 1.79% and 1.44–1.75% with mean 1.60% for high and low yielding trees, respectively. However, variations in temperature, rainfall patterns, and extreme weather events affect mango cultivation, affecting flowering, fruit set, and quality (Halder et al., 2024). Anon (2017) presented leaf levels for N in different mango cultivars in Australia as being in the range of 1.0 to 1.5%. The variation of leaf nitrogen in the selected orchards (two seasons) of the selected mango trees cv. Timor is depicted in Fig. 2, curve a.

Compared to other plant minerals, phosphorus has a far greater impact on the natural and agroecosystem (Brady & Wei, 2002). Phosphorus is regarded as an essential component of several plant compounds that are involved in respiration, photosynthesis, cell division, and growth (Khan & Ahmed, 2020). The results show that the leaf phosphorus range was 0.15–0.39% with an average of 0.26% ± 0.07% in the OFF season and 0.11–0.33% with an average 0.21 ± 0.07% in the ON season (Table 2) and with a comparison with the results of Reddy et al. (2001), who presented mean leaf nutrients of phosphorus form different orchards irrespective of variety (1995–1997) of 0.06–0.24% with an average 0.13% and 0.06–0.22 with an average 0.11% for high and low yielding trees, respectively. The variation of leaf phosphorus in the selected orchards (two seasons) of the selected mango trees cv. Timor is depicted in Fig. 2, curve b.

Potassium is a vital nutrient in mango production (González-Acuña et al., 2009). It significantly affects the growth and development of mango trees, particularly during the early stages of growth when young mango trees need potassium (K) for quick girth and branching development. The results show that the leaf potassium range was 0.55–0.99% with an average of 0.82 ± 0.13 in the OFF season and 0.45–0.96% with an average of 0.77 ± 0.16% in the ON season (Table 2). In comparison with the results of Reddy et al. (2001), who presented mean leaf nutrients of potassium from different orchards irrespective of variety (1995–1997) of 67–0.90% with an average of 0.78% and 0.67–0.85% with an average 0.75% for high and low yielding trees, respectively. Anon (2017) presented leaf levels for K in different mango cultivars in Australia as being in the range of 0.7 to 1.2%. The variation of leaf K in the selected orchards (two seasons) of the selected mango trees cv. Timor is depicted in Fig. 2, curve c. However, as seen in Table 3, the K concentration in mango leaves ranged between 0.008 to 0.95% as stated by previous studies.

**Table 3:** The mineral range of mango leaves from different research papers.

**Mineral** **composition**	**Range of composition** ** (%)**	**References**
Nitrogen	0.003–2.60	Abou-Awad, Al-Azzazy & Afia (2012), Princwill-Ogbonna, Ogbonna & Ogujiofor (2019), Ali et al. (2020)
Phosphorus	0.007–0.48	Okwu & Ezenagu (2008), Abou-Awad, Al-Azzazy & Afia (2012), Rymbai et al. (2013), Laulloo et al. (2018), Princwill-Ogbonna, Ogbonna & Ogujiofor (2019), Ali et al. (2020)
Potassium	0.008–0.95	Okwu & Ezenagu (2008), Abou-Awad, Al-Azzazy & Afia (2012), Princwill-Ogbonna, Ogbonna & Ogujiofor (2019), Ali et al. (2020)
Calcium	0.003–4.41	Okwu & Ezenagu (2008), Abou-Awad, Al-Azzazy & Afia (2012), Rymbai et al. (2013), Laulloo et al. (2018), Princwill-Ogbonna, Ogbonna & Ogujiofor (2019), Ali et al. (2020)
Magnesium	0.009–1.58	Okwu & Ezenagu (2008), Abou-Awad, Al-Azzazy & Afia (2012), Princwill-Ogbonna, Ogbonna & Ogujiofor (2019), Ali et al. (2020)

The results show that the leaf calcium concentration range was 1.94–2.73% with an average of 2.33 ± 0.25% in the OFF season and 1.92–2.57% with an average of 2.22 ± 0.20% in the ON season (Table 2), however, calcium (Ca) concentration in mango leaves is in the range between 0.003 and 4.41% as depicted in Table 3, as reported by previous studies. One of the most important elements for the growth and development of plants is calcium. Plant damage can result from high and low calcium levels on pertinent physiological and biochemical processes (Guo et al., 2023). The variation of leaf calcium in the selected orchards (two seasons) of the selected mango trees cv. Timor is depicted in Fig. 2, curve d.

**Figure 3:**
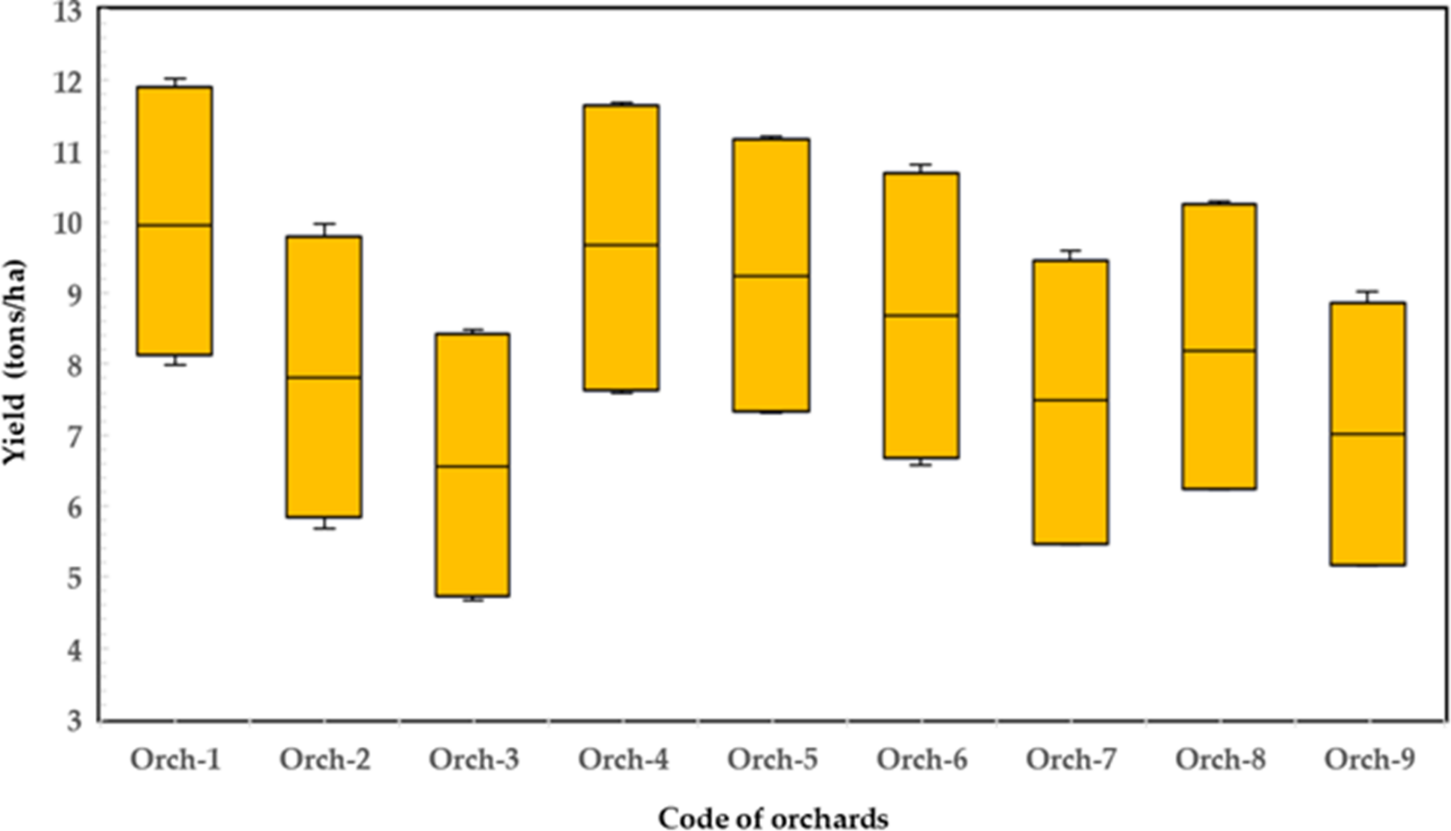
Variation of Timor mango yield with nine orchards for the combined two seasons (data collected from nine orchards).

An effective method for determining the present nutrient levels in an orchard is the chemical analysis of mango leaves. Leaf chlorosis was influenced by the content of magnesium (Mg). It is an essential component of chlorophyll and is involved in photosynthesis. High concentrations can produce dark green fruit in mangoes (Poffley & Owens, 2005). Magnesium deficiency can be distinguished by the interveinal chlorosis of the old leaves down the trees. The results show that the leaf magnesium range was 0.26–0.80% with an average of 0.56 ± 0.17% in the OFF season and 0.21–0.75% with an average of 0.47 ± 0.18% in the ON season (Table 2). In another study, Zuazo, Pleguezuelo & Tarifa (2006), magnesium leaf concentrations of mango cv. Osteen and cv. Keitt were in the range of 0.16–0.21%. However, Table 3 shows the mineral composition of mango leaves as reported by previous studies. The variation of leaf magnesium in the selected orchards (two seasons) of the selected mango trees cv. Timor is depicted in Fig. 2, curve e. The variation of leaf magnesium of selected mango trees (cv. Timor) orchards (two seasons) is depicted in Fig. 3. Joshi et al. (2016) stated differences in leaf nutrients (N, P, and K concentration) of mango trees. The higher leaf N, P, and K status was owing to the higher accessibility of these nutrients in the soil of Reddy et al. (2001).

Leaf analysis is useful for identifying imbalances and supporting fruit fertilizer recommendations. Because of the strong relationship between nutrient availability in the soil and leaf nutrient concentration, leaf analysis can be used to assess the fertility of the soil (Faria et al., 2016). This study can assist in recommending fertilizers at the right moment to address nutritional deficiencies, particularly phenological cycle stages. Leaf chlorophyll (Chl *a* and Chl *b*) is a vital pointer for evaluating the photosynthetic size, plant senescence, environmental stress, and nitrogen (N) rank of leaves (Darvishzadeh et al., 2008; Yu et al., 2014; Houborg et al., 2015; Croft et al., 2020; Zhu et al., 2020). Leaf chlorophyll plays an important part in catching photons and moving electrons through the development of photosynthesis (Porcar-Castell et al., 2014; Zhou et al., 2020). The results show that the leaf Chl *a* range was was 1.13–1.92 mg/g FM with an average of 1.56 ± 0.25 mg/g FM in the OFF season and 1.14–1.65 mg/g FM with an average of 1.39 ± 0.16 mg/g FM in the ON season (Table 2). The results show that the leaf Chl *b* range was 0.61–1.31 mg/g FM with an average of 0.96 ± 0.23 mg/g FM in the OFF season and 0.62–1.23 mg/g FM with an average of 0.92 ± 0.19 mg/g FM in the ON season (Table 2). The leaf nutrient status signals the healthy grade of tree vigor. The environmental variables show a significant and essential role in initiating mango flowering (Kumar et al., 2013).

A crop’s growth and development, as well as its fitness for producing a sufficient yield, depend on its favorable carbohydrate status. The distribution of carbohydrates to the roots for growth and storage, which guarantees a favorable energy status for the crop the following season, depends on the leaf’s carbohydrate state (Glanz-Idan & Wolf, 2020; Aluko et al., 2021). The synthesis of the floral stimulus in mango trees is somehow linked to the accumulation of carbohydrates in the leaves (Kumar et al., 2013). Carbohydrates are important in flower growth and fruit setting (Prasad et al., 2014). The results show that the leaf total carbohydrates fraction range was 9.92–25.28% with an average of 17.31 ± 4.96% in the OFF season and 6.40–20.48% with an average of 12.58 ± 4.57% in the ON season (Table 2). The highest carbohydrate content of the leaf at the harvesting stage (19.85%) was seen in cv. Neelum during the main season (Kumar et al., 2013). Shaban et al. (2019) reported that the values of the carbohydrate content of the leaf (10.60 and10.27%) for the ON and OFF seasons, respectively, were observed during the control treatment. The balance between vegetative and reproductive growth is linked to the plant carbohydrate: nitrogen ratio (Nafees et al., 2013). Mango trees’ ideal nitrogen status could be maintained to attain this balance. However, the carbohydrate: nitrogen ratio in the leaf was derived by dividing the total carbohydrate content by the total nitrogen content (Kumar et al., 2013).

### Mango yield variability with leaf nutrient concentration

The mean yields for the ON and OFF seasons are depicted in Table 2. The variation between seasons is high due to the phenomena of ”ON season” and OFF season (Elkhishen, 2015). The ON season gave a big yield, and the OFF season gave a light yield. Results indicated that for fruit yield per plant from OFF season was less compared to the main season. This may be attributed to poor competition for nutrients among the developing fruits, which act as a sink, besides fluctuating environmental conditions during the OFF season, compared to the main season (Shivu Prasad et al., 2015). Furthermore, according to Sharma et al., (2019), in biennial bearing, a big yield is followed by a light yield the next season. “OFF” and “ON” seasons are often linked to cycles in the stores of carbohydrates (Rossouw et al., 2024). An uneven fruiting pattern across the seasons is a sign of irregular bearing (Shivashankara & Mathai, 2000; Smith & Samach, 2013; Sharma et al., 2020). However, mangos bear irregularly, alternately, or biennially, depending on the location, agronomic methods, and cultivar (Sudha, Balamohan & Soorianathasundaram, 2012; Torgbor et al., 2023). In the study of Kumar et al. (2014), they observed that fruit set (%) was reduced in mango cultivars in the main season compared to the OFF season.

Figure 3 shows the variation of mango yield in the orchard for two seasons. It is obvious from the data that yields differ among orchards due to agricultural practices. However, the variation in mango production across seasons may be caused by a variety of factors, including weather, management, biennial bearing, nutrient use/carbohydrate storage, *etc*., according to Torgbor et al. (2023). Most of these factors were not involved in our ANN model to predict the mango yield of the Timor cultivar, as these factors were outside the scope of this preliminary study. The novelty of our approach is in its ability to use leaves, nutritional status and an ANN model on out-of-season commercial yield data. The timing and location of plant sampling vary depending on the plant species and the conditions under which the plant is grown, and other factors. According to Chadha, Samra & Thakur (1980), sampling leaves from 6–7 months old branches four weeks before harvest is appropriate for prediction purposes for mangoes to avoid and restrict the effect of leaf age and leaf sampling site. Therefore, the variability is attributed to other factors such as soil nutrient availability, soil type, growing conditions, and relevant physiological and biochemical processes associated with each orchard, which can influence fruit quality and yield. Moreover, quantifying the degree of nutrient depletion in various plant tissues (leaves, roots, and fruits) during “ON” years and how this differs among fruit species and growth environments should be the main focus of future research.

**Figure 4:**
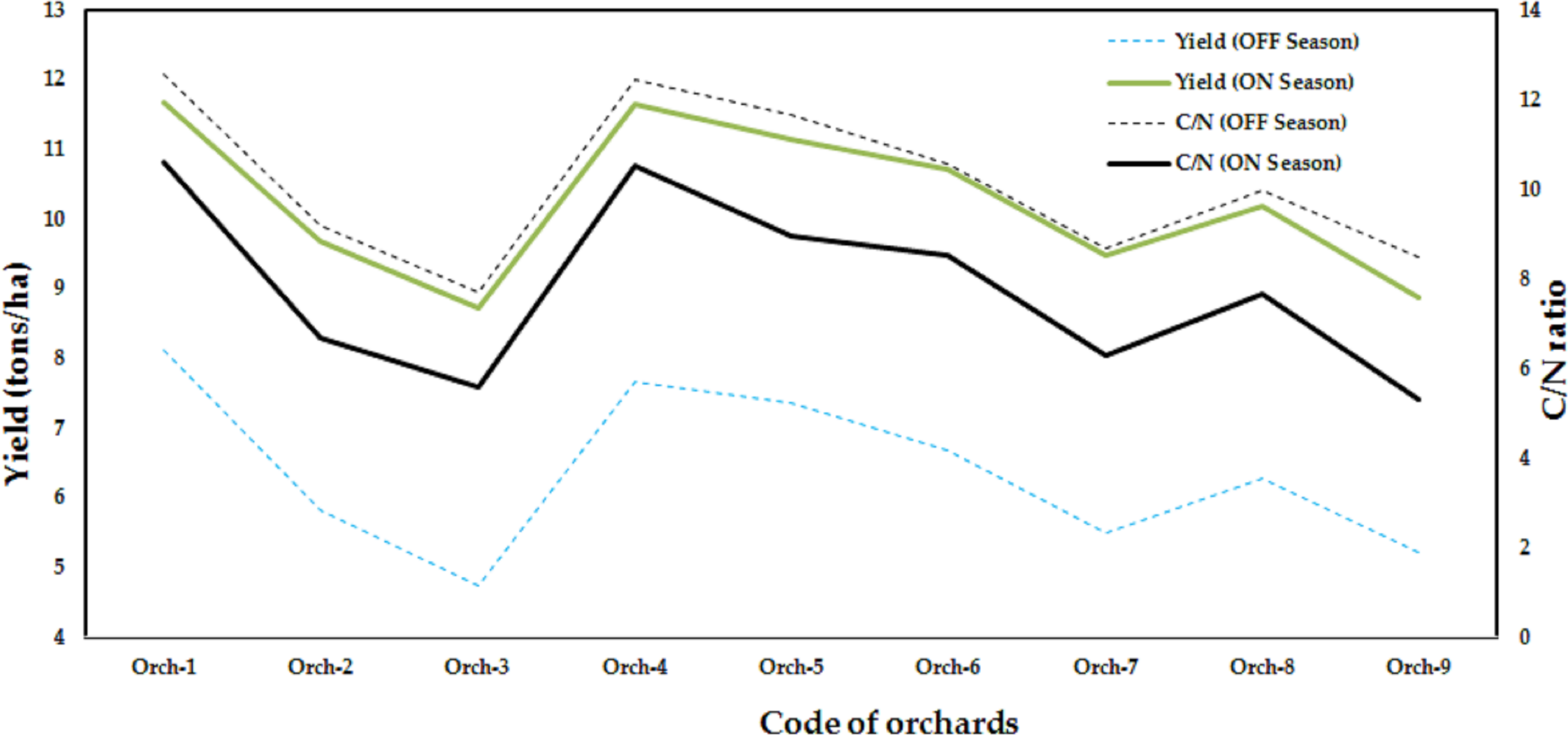
Variation of the overall average of Timor mango yield and the carbohydrate: nitrogen (C/N) ratio with nine orchards for two seasons (data collected from nine orchards).

The data in Fig. 3 make it clear that agricultural techniques cause yields to vary among orchards. However, according to Torgbor et al. (2023), various factors, including weather, management, biennial bearing, nutrient use/carbohydrate storage, *etc*., may contribute to the variation in mango yields obtained in different seasons. Since they were outside the purview of our exploratory study, the majority of these parameters were not included in our ANN model to forecast the Timor cultivar’s mango production. Our method’s capacity to apply the ANN model and leaf nutrition status to out-of-season commercial yield data makes it distinctive. Figure 4 shows the variation in the average mango yield and the carbohydrate-to-nitrogen ratio (C/N) in the orchards for two seasons. According to Sudha, Balamohan & Soorianathasundaram (2012), the C/N ratio had a value of 10.8 as a result of using different foliar sprays of nitrogenous chemicals, however, the changes in C/N ratio had a greater effect on fruit set and yield. The highest C/N ratio of the leaf at the harvesting stage was found to be 10.34 in cv. In Alphonso, during the OFF season and for the main season, the C/N ratio of the leaf at the harvesting stage was recorded at 10.25 (Porcar-Castell et al., 2014). However, environmental variables and seasonal variations in the biochemical composition, specifically, the amount of nitrogen and carbohydrates, may be to blame for this. It seems that the C/N ratio depended on the rootstock (Vittal et al., 2023). According to Shaban et al. (2019), the control treatment had a C/N ratio of 8.48 for the first season and 8.15 for the second. Nonetheless, the maximum blossom percentage agreed with a C/N ratio of 10.

**Figure 5:**
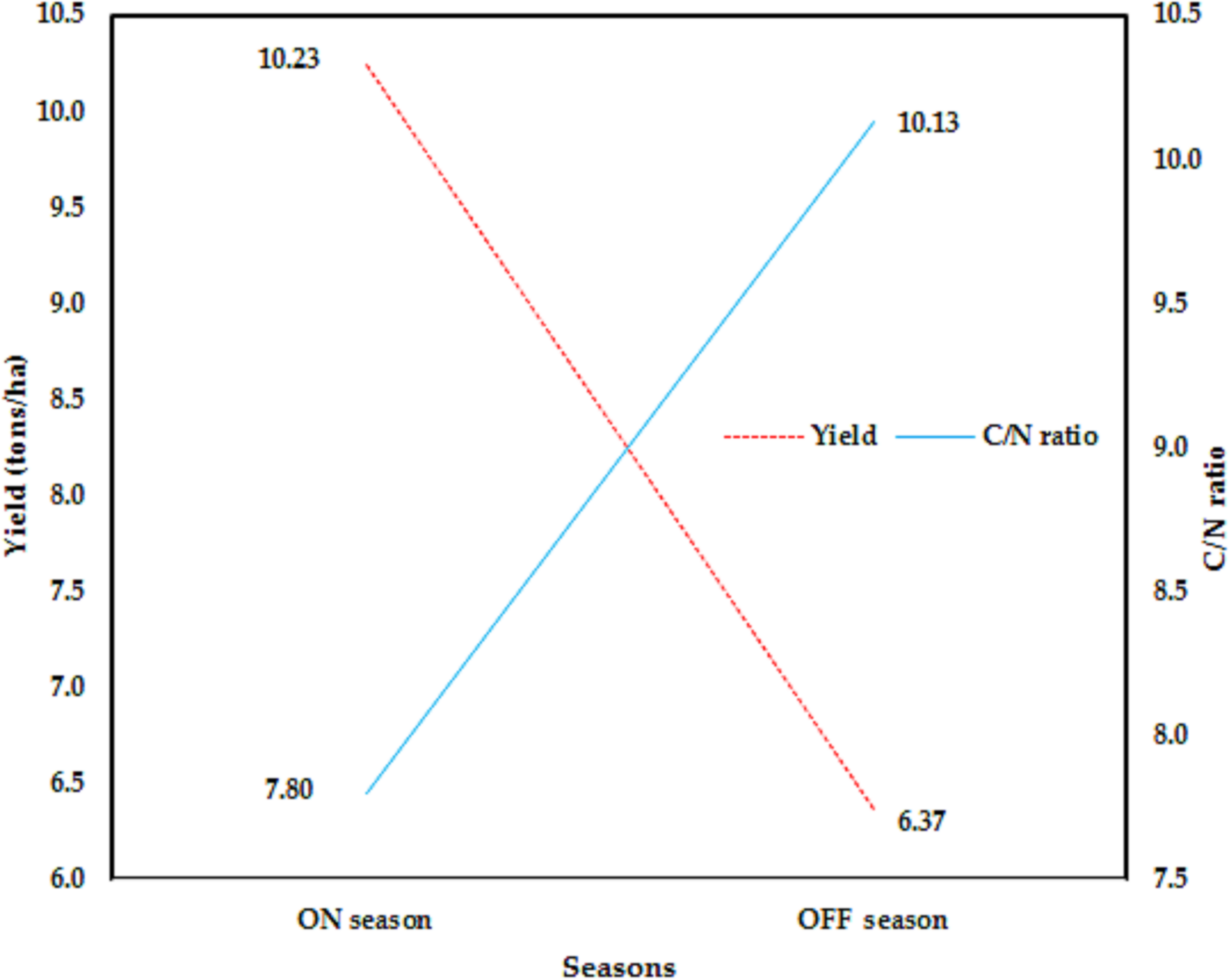
The relationship between the C/N ratio and yield, as the overall average, across the growing season (data collected from nine orchards).

In this research work, at the harvesting time, the relationship of the C/N ratio and yield, as the overall average, against the growing season, is shown in Fig. 5. It is clear that the opposite trend exists between carbohydrate-to-nitrogen ratio and yield at the harvesting time. Since the fruit is a sink of nutrients, including carbohydrates, in the ON season, it happens from the beginning of the holding fruits that the fruit withdraws a large amount of carbohydrates from leaves until harvest, consequently, at harvest at the end of the season, the amount of carbohydrates in the leaves drops. The opposite occurs in the light season, OFF season, as a result of the crop being low, so the amount of carbohydrate withdrawal is less, and thus the level of carbohydrates in the leaves at harvest time is high.

Table 4 indicates Pearson’s correlation coefficients for the various features, which were determined using SPSS software, Version 26, to understand the relationship between the variables. As shown in Table 5, the yield had a positive correlation with all parameters with a low correlation (*p* < 0.01). This pattern was noted in a different study by Khoshnood & Torkashvand (2016), who found that the N, K, Ca, Mg, and N/Ca ratio in leaves and kiwi yield had significant correlation coefficients of 0.386, 0.270, 0.235, 0.215, and 0.355, respectively. Chl *a* was positively correlated with both leaf N and leaf P (*p* < 0.01) (Lia et al., 2018).

**Table 4:** Pearson’s correlation coefficients between various features.

**Nutrition elements**	**N**	**P**	**K**	**Ca**	**Mg**	**Chlorophyll content a**	**Chlorophyll content b**	**Total carbohydrates fraction**	**Yield**
N	1	0.976^**^	0.946^**^	0.990^**^	0.986^**^	0.983^**^	0.976^**^	0.968^**^	0.244^**^
P		1	0.916^**^	0.977^**^	0.978^**^	0.963^**^	0.949^**^	0.974^**^	0.198^**^
K			1	0.925^**^	0.959^**^	0.905^**^	0.943^**^	0.897^**^	0.305^**^
Ca				1	0.980^**^	0.977^**^	0.979^**^	0.969^**^	0.273^**^
Mg					1	0.961^**^	0.973^**^	0.963^**^	0.274^**^
Chlorophyll content a						1	0.940^**^	0.973^**^	0.124
Chlorophyll content b							1	0.916^**^	0.426^**^
Total carbohydrates fraction								1	0.058

**Notes.**

** Correlation is significant at the 0.01 level (1-tailed).

**Table 5:** Comparison of the conventional ANN model’s (8-20-1) performance on the training and testing datasets using statistical measures.

**Output node**	**Training dataset**				**Testing dataset**			
	**RMSE** **(ton/ha)**	**MAE** **(ton/ha)**	**MAPE (%)**	**R** ^**2**^	**RMSE** **(ton/ha)**	**MAE** **(ton/ha)**	**MAPE (%)**	**R** ^**2**^
Mango yield	0.262	0.209	2.621	0.989	0.229	0.183	3.021	0.975

**Figure 6:**
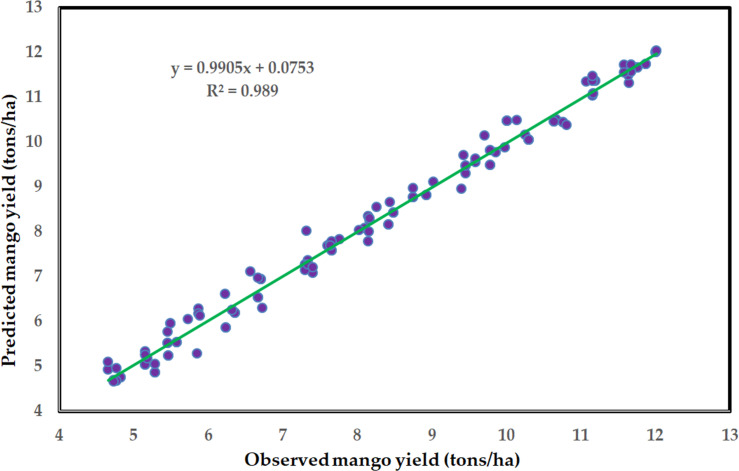
Scatter plot of observed Timor mango yield values compared to estimated values by the ANN model using the training dataset with regression line.

### Performance of predictable ANN model for mango yield

Table 5 displays the error outcomes of the built ANN model, which compares the mean absolute error (MAE), root mean square error (RMSE), and mean absolute percentage error (MAPE), during the training and testing stages. These findings indicate that the ANN model accurately predicted the dependent variable of mango yield based on the examined explanatory variables. A scatter curve of the observed mango yield values compared to the values the ANN model predicted during training is displayed in Fig. 6. However, the quality of the input data used in a model’s training and testing is a major factor in determining its strength (Sharma et al., 2020). Variations in agricultural methods may be the primary cause of the mistakes associated with the ANN model created in this study, especially at the field level. However, the ANN technique is the most applied algorithm for crop yield prediction (Shawon et al., 2025). Generally, in agriculture, the amount of crop production estimation is crucial; industrialists, producers, and consumers all assist by perceiving the early yield (Neethi & Raviraj, 2025).

**Figure 7:**
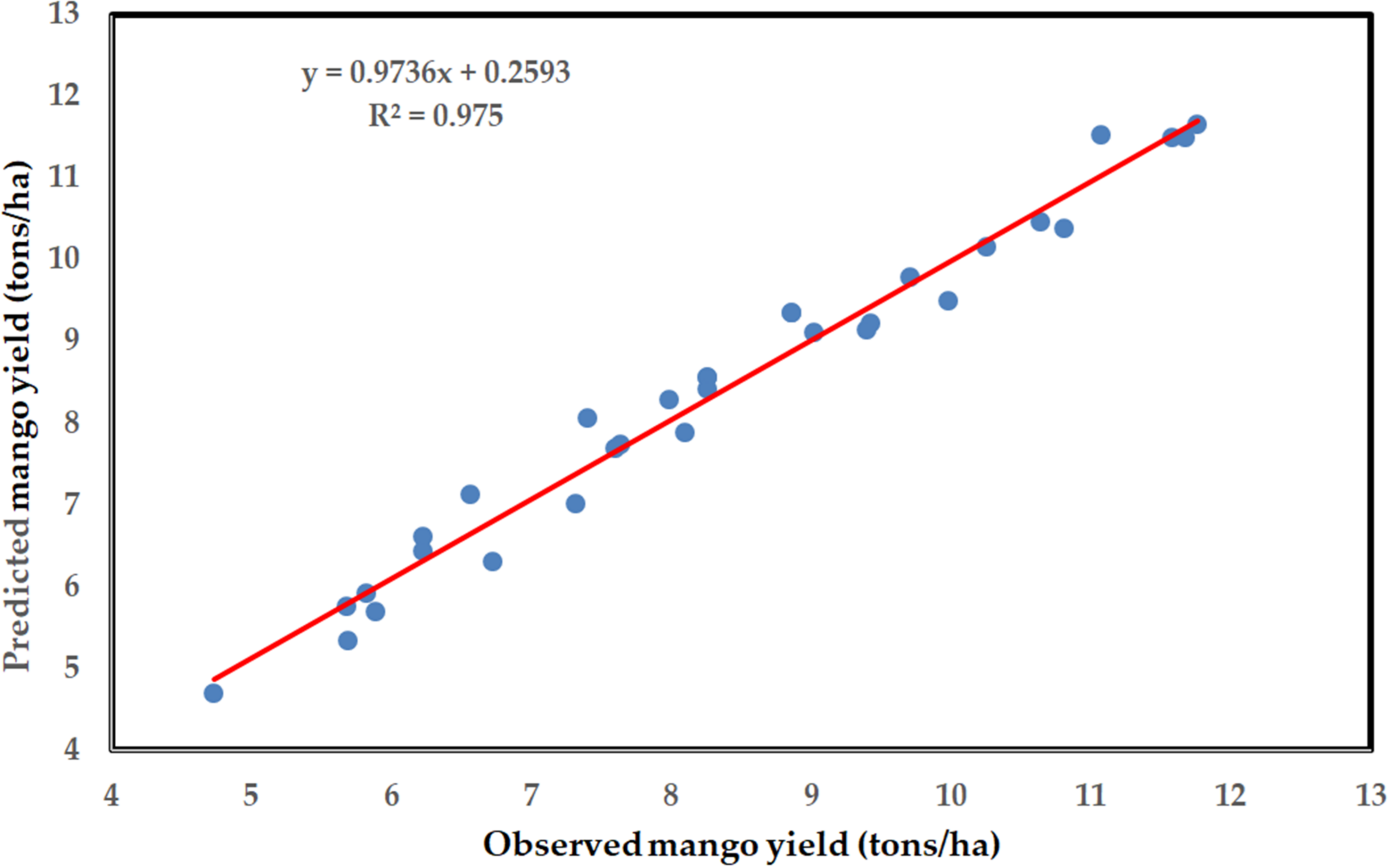
Scatter plot of observed Timor mango yield values compared to estimated values by the ANN model using the testing dataset with regression line.

A scatter plot of the observed mango yield values compared to the values that the ANN model predicted during the testing phase is displayed in Fig. 7. The estimation findings over the data range were correct, as indicated by the values of R^2^, MAE, and RMSE between the measured and estimated values of mango yield in Table 5. However, the data points in the scatter plots are not dispersed around the associated regression lines, Figs. 6 and 7 show that the prediction of mango yield is partially reliable. Nonetheless, Table 5 indicates that the MAPE values were below 10%, which is deemed acceptable (Qazi et al., 2015).

### Contribution of each input parameter to the prediction of mango yield using the developed ANN model

**Figure 8:**
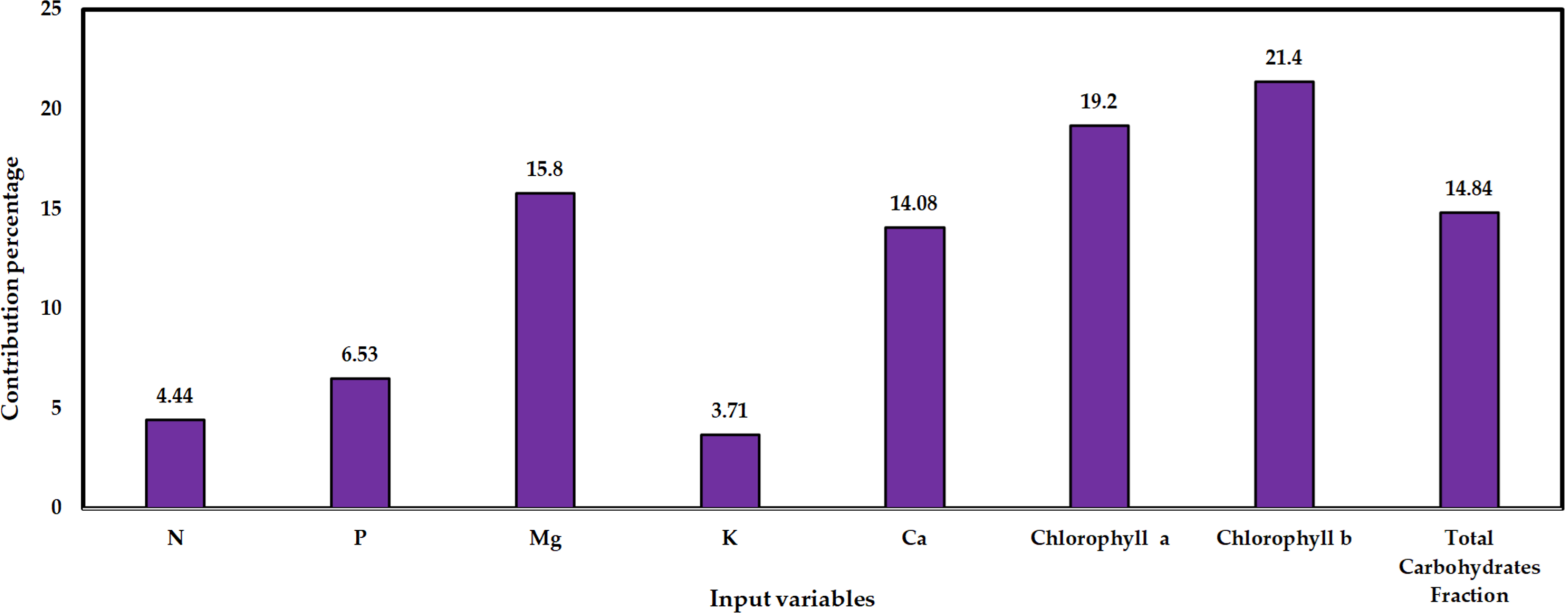
Relative importance is the contribution of input variables to the prediction of Timor mango yield using the established ANN model of (8-20-1).

Each input parameter’s proportional importance as a percentage of its overall contribution is displayed in Fig. 8. Remember that when a parameter has high sensitivity, even minor adjustments can significantly affect system performance, and vice versa. N concentration, a process input variable, has a contribution percentage of 4.44%, P concentration contributed by 6.53%, Mg concentration contributed by 15.9%, K concentration contributed by 3.71%, Ca concentration contributed by 14.08, Chl *a* contributed by 19.2%, Chl *b* contributed by 21.4%, and total carbohydrates friction contributed by 14.84% on mango yield prediction as depicted in Fig. 8.

## Conclusions

A vital component of the agricultural economy, mango cultivation creates jobs and revenue in various localities. Harvesting, planning and logistics are optimized by accurate production estimation; manual approaches are typically ineffective and prone to mistakes. Thus, the conclusions of this investigation displayed that the established ANN model presented better accuracy (R^2^ value of 0.989) was achieved using the training and testing datasets, respectively. The ANN model is well appropriate for predicting mango yield and presents succinct training, even with a limitation in the amount of data, when completely using the leaf nutrition characteristics of the mango. This limitation is overcome when the data are combined into the model since most of the difference in productivity is because of these characteristics. Moreover, the ANN model can improve decisions on future harvest and marketing logistics and enable the industry to maximize production and reduce food waste. The yield for other areas and the country as a whole might be predicted using the proposed method again. Chl *a* and Chl *b* had the highest contribution by 19.2% and 21.4%, respectively, on mango yield prediction. Based on our findings, we offer several important implications for researchers and mango growers, as nutrition is the most important among the many problems affecting growth and fruit yield, and agricultural practices cause yields to vary among orchards. Only leaf nutrient data were used in the developed ANN model to predict mango yield, but there are factors like climate, irrigation, pruning, and biotic stress that significantly influence mango yield, and we propose the inclusion of such variables in future models. Lastly, the possibility that this approach could yield reliable estimates for other perennial tree crops could be investigated in future studies.

## Supplemental Information

10.7717/peerj.20013/supp-1 Supplemental Information 1 The raw measurements

## Additional Information and Declarations

### Data Availability

The following information was supplied regarding data availability: The raw measurements are available in the Supplementary File 1. We used a graphical user interface simulation software called Qnet 2000. However, this simulation software does not offer codes, but it provides interactive screens (Figure 1) like SPSS software (IBM Corporation, New York, NY, USA). Also, Qnet 2000 is a Shareware software in the category Miscellaneous developed by Qnet 2000 https://qnetv2kt.software.informer.com/.
